# Physical exercise in brain tumor patients: a systematic review

**DOI:** 10.3389/fonc.2026.1810188

**Published:** 2026-09-07

**Authors:** Arooba Iqbal, L. Nicolas Gonzalez Castro

**Affiliations:** 1 Department of Medicine, Dow University of Health Sciences, Karachi, Pakistan; 2 Center for Neuro-Oncology, Dana-Farber Cancer Institute, Boston, MA, United States; 3 Center for Tumors of the Nervous System, Mass General Brigham, Boston, MA, United States; 4 Department of Neurology, Mass General Brigham, Boston, MA, United States; 5 Department of Neurology, Harvard Medical School, Boston, MA, United States

**Keywords:** brain tumor (BT), glioblastoma, glioma, physical exercise (EX), quality of life

## Abstract

**Background:**

Brain tumors – both primary and metastatic – are a leading cause of mortality and disability among cancer patients. For patients with cancer, physical exercise has proven effective in the management of multiple cancer- and treatment-related symptoms. However, the benefits of physical exercise have been understudied in patients with a brain tumor diagnosis.

**Methods:**

We systematically reviewed the literature on physical exercise interventions in patients with brain tumors. A systematic search of PubMed and the Cochrane Library was conducted up to December 2025 following PRISMA guidelines. Studies were screened according to predefined inclusion and exclusion criteria, and data were extracted using a structured approach. Study quality and level of evidence were assessed using the Oxford Centre for Evidence-Based Medicine (CEBM) Levels of Evidence, and risk of bias was evaluated using the Cochrane Risk of Bias 2 (RoB 2) tool for randomized controlled trials (RCT) and Risk Of Bias in Non-randomized Studies of Interventions (ROBINS -I) for non-randomized studies. Results were synthesized descriptively.

**Results:**

Thirty-one studies (23 clinical trials, 4 observational studies, 2 qualitative analyses, 1 case report and 1 case study) met inclusion criteria and were synthesized descriptively to accommodate for the heterogeneity of study designs and recorded outcomes. Brain tumor patients were able to participate in aerobic, strength training, mixed (aerobic + strength), yoga and balance exercise interventions, and the findings suggest potential improvements in physical and cognitive function as well as quality of life. Adverse events reported were rare and consistent with exercise-related adverse events reported for individuals without cancer.

**Conclusions:**

The available literature suggests the feasibility and overall safety of physical exercise in brain tumor patients although evidence of clinical effectiveness remains preliminary. The majority of findings are based on structured intervention studies, and broader applicability remains to be determined due to the limited evidence available. The high risk of bias noted in most studies, as well as their generally small sample sizes and diversity of exercise interventions studied, highlight the need for larger prospective clinical trials to determine clinical effectiveness and inform the design of exercise guidelines for brain tumor patients.

**Systematic review registration:**

https://www.crd.york.ac.uk/PROSPERO/view/CRD420251111272, identifier CRD420251111272.

## 1 Introduction

A brain tumor diagnosis is a life-changing event. Despite decades of research leading to incremental therapeutic advances, aggressive primary and metastatic brain tumors remain a major cause of morbidity and mortality among cancer patients. After their initial diagnosis and treatment, brain tumor patients experience progressive worsening of their physical and cognitive abilities due to multiple factors. Standard treatments including surgery, radiotherapy and chemotherapy are known to cause long-term cognitive changes and affect problem-solving abilities (1–3) while motor skills, complex movements and balance are often affected depending on the type, location and size of the tumor, ultimately affecting patients’ quality of life (QoL) and overall survival (4). Although the literature on the effectiveness of exercise in brain tumor patients is still limited, the more abundant literature in other tumor types provides evidence that physical exercise modalities such as aerobic exercise and strength training can be effective in reducing cancer specific symptoms such as fatigue, as well as improving motor function, cognitive abilities, QoL, and in some instances, overall survival (5–7). Understanding and acknowledging the significant impact of exercise and a healthy lifestyle, major academic societies including the American Society for Clinical Oncology (ASCO), the American Cancer Society (ACS), and the American College of Sports Medicine (ACSM) have developed FITT (Frequency, Intensity, Time, and Type)-based exercise guidelines for cancer survivors broadly, outlining recommendations regarding the duration, type, and intensity of exercise. However, guidance specifically addressing the unique clinical context of brain tumor patients remains limited, highlighting the importance of the present review (8–10).

Additionally, physical exercise has been found to induce a range of physiological changes providing mechanistic clues that may explain the noted improvements in patients’ neurological and physical functioning. Exercise activates key metabolic and signaling pathways, including AMPK and PGC-1α, promoting mitochondrial biogenesis and improved metabolic regulation. It also modulates immune function and inflammatory signaling through the release of myokines, which may contribute to reduced chronic inflammation and enhanced physiological resilience. In oncology, these systemic adaptations may influence tumor growth, metabolic reprogramming, and treatment responsiveness, providing a biological rationale for investigating exercise in brain tumor patients (11–14).

The aim of this systematic review is to summarize the available literature on the impact of physical exercise on the overall health of brain tumor patients including its effect on motor function, sleep, mood, cognition, and overall QoL, as well as review the available data on the effect of exercise on tumor progression, symptom progression and overall survival. Both primary brain tumors and brain metastases were included, as patients in these groups share overlapping functional impairments and treatment-related challenges, making exercise interventions relevant across tumor types. Throughout, we highlight the feasibility and adherence of patients to prescribed exercise regimens in the reviewed studies. Our goal is to summarize the evidence and uncover the gaps in the literature to guide future research, with the hope of informing the development of brain tumor-specific exercise guidelines.

## 2 Methods

### 2.1 Search strategy

A systematic literature search was performed in MEDLINE (via PubMed) and the Cochrane Library according to PRISMA guidelines, following the PRISMA 2020 checklist, as these databases provide comprehensive coverage of peer-reviewed biomedical literature and evidence-based medicine based on high-quality clinical trials. We did not query other databases that are generally preferred for pharmacology and medical device studies, as these were not relevant to our review topic (15). This systematic review protocol was prospectively registered in PROSPERO (registration no; CRD420251111272). Studies examining the effect of exercise on brain tumor patients were identified. The search strategy employed was (brain neoplasms OR brain tumor OR CNS tumor OR glioma OR high-grade glioma OR glioblastoma OR IDH-mutant glioma OR metastases OR meningioma OR primary CNS lymphoma OR PCNSL) AND (exercise OR physical exercise OR yoga OR physical activity OR ski-based exercise) for both PubMed and Cochrane Library databases, capturing all results until December 9, 2025. Our search strategy was designed to capture the entire spectrum of physical exercise interventions and their effect on all types of brain tumors including primary and metastatic tumors.

### 2.2 Study selection

Records were identified through database searching. Screening was conducted independently by two reviewers using a two-stage process. In the first stage, records were excluded based on title screening, followed by the exclusion of records after abstract review. Remaining articles underwent full-text assessment for eligibility. Following full-text review, studies involving only structured or symptom-focused rehabilitation interventions, as well as behavioral therapies (e.g., psychotherapy or cognitive behavioral therapy), were excluded. Additionally, studies with non-relevant populations or unavailable results were excluded. Any disagreements during the screening process were resolved through discussion and consensus between the reviewers.

### 2.3 Inclusion and exclusion criteria

Inclusion criteria for our systematic review included studies published before December 9, 2025 in English, evaluating the clinical outcomes of physical exercise interventions in brain tumor patients, including both adult and pediatric populations. Age restrictions were not applied during study selection. For the purpose of this review, *exercise interventions* were defined as planned, structured, and repetitive physical activities performed with the objective of improving or maintaining physical fitness or health outcomes, consistent with standard definitions in exercise science. These interventions included modalities such as aerobic exercise (e.g., walking, running, cycling, skiing), mind–body exercise (e.g., yoga), or other structured physical training programs delivered during, or after brain tumor treatment. Exercise interventions could be supervised or unsupervised, provided they involved a clearly defined physical activity program with specified frequency, duration, or intensity. Study designs included clinical trials, observational studies, case series and case reports. Due to the limited availability of high-quality studies in this area, a broad range of study designs were included to comprehensively capture the existing evidence. Studies including mixed cancer populations were also included if the sample included brain tumor patients. Overall outcomes were extracted when brain tumor-specific results were not reported separately. Studies in languages other than English, reviews, meta-analyses or studying the effect of rehabilitation interventions were excluded when the primary focus was multidisciplinary functional restoration or therapeutic management of neurological deficits (e.g., physiotherapy, occupational therapy, or comprehensive neurorehabilitation programs). Studies primarily evaluating non-exercise-based cognitive, behavioral, or rehabilitative therapies were also excluded.

### 2.4 Data extraction

Data extraction from eligible studies included: author(s), publication year, study design, patient population, brain tumor type, treatment, sample size, type of physical exercise intervention, duration of intervention, outcome(s), major findings, class of evidence, adverse events and limitations. The primary outcomes of interest were physical fitness (including aerobic capacity/cardiorespiratory fitness, strength, functional abilities), cognitive performance, tumor specific symptoms (including but not limited to coordination dysfunction, anxiety, depression, weakness, headache, sleep disturbances, range of motion, pain, fatigue) and QoL. Additional outcomes related to intervention feasibility, adherence, and safety were also extracted and reported when available.

### 2.5 Assessment of level of evidence

We categorized the articles based on study type and their eligibility for inclusion in the systematic review was evaluated based on the population, intervention, control, outcomes and study design (PICOS) criteria. The strength of the evidence for all the studies included was evaluated using the Oxford Centre for Evidence-Based Medicine (CEBM) Levels of Evidence (16). For the randomized controlled trials reviewed, risk of bias was formally assessed using the Cochrane Risk of Bias 2 (RoB 2) tool (17). Risk of bias was also assessed for non-randomized studies using the Risk Of Bias In Non-Randomized Studies of Intervention (ROBINS-I) tool.

### 2.6 Data synthesis

Results were synthesized descriptively in the form of a data extraction table highlighting each article’s key characteristics. The table was designed focusing on the impact of physical exercise interventions on different outcomes in patients with brain tumors. For statistical measures including percentages (such as feasibility, adherence, improvement muscle strength, etc.), percent increase and median values with *p*-value and confidence intervals were noted where applicable.

## 3 Results

**Figure 1:**
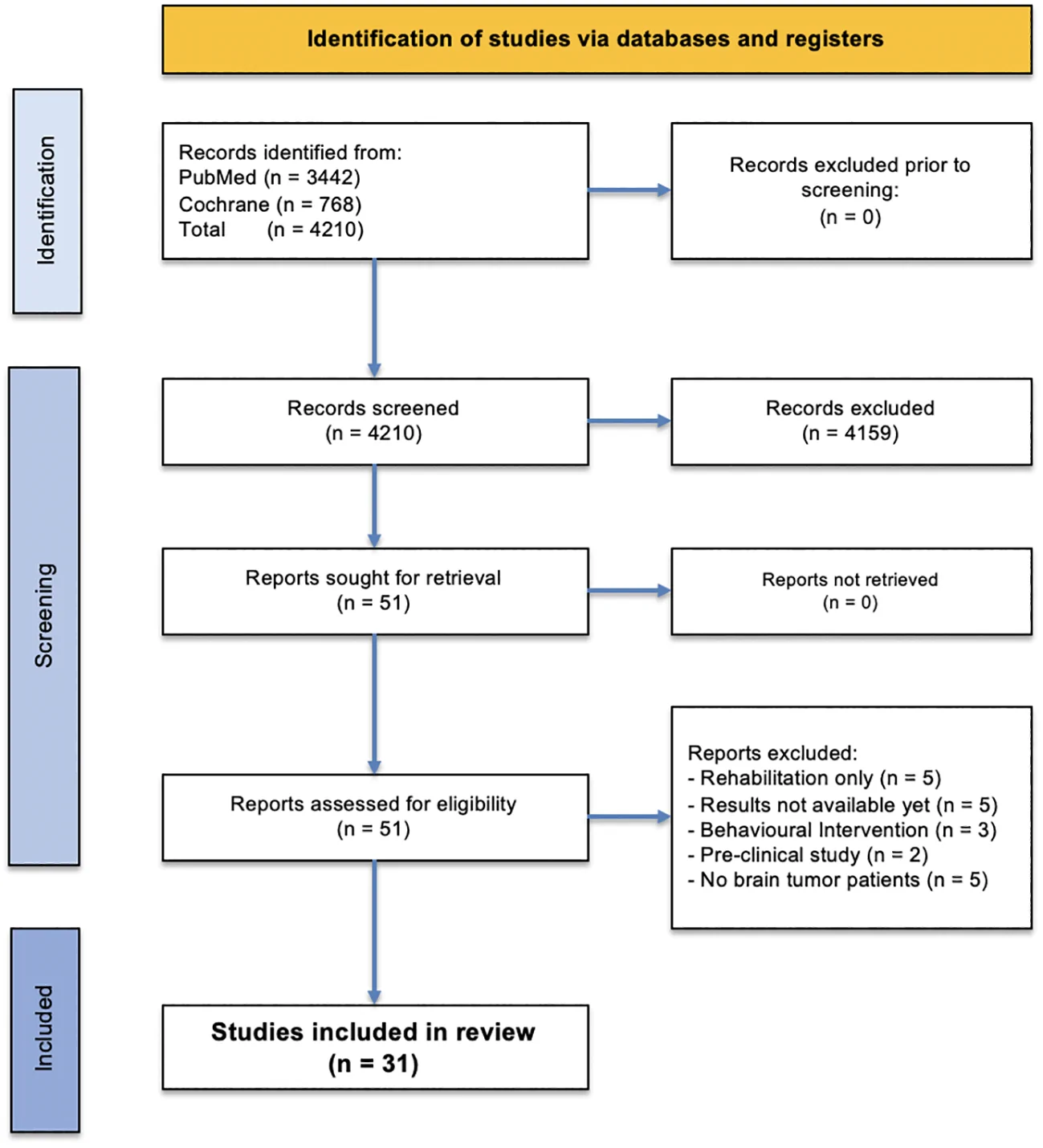
Articles identified, screened and included in the systematic review.

**Figure 2:**
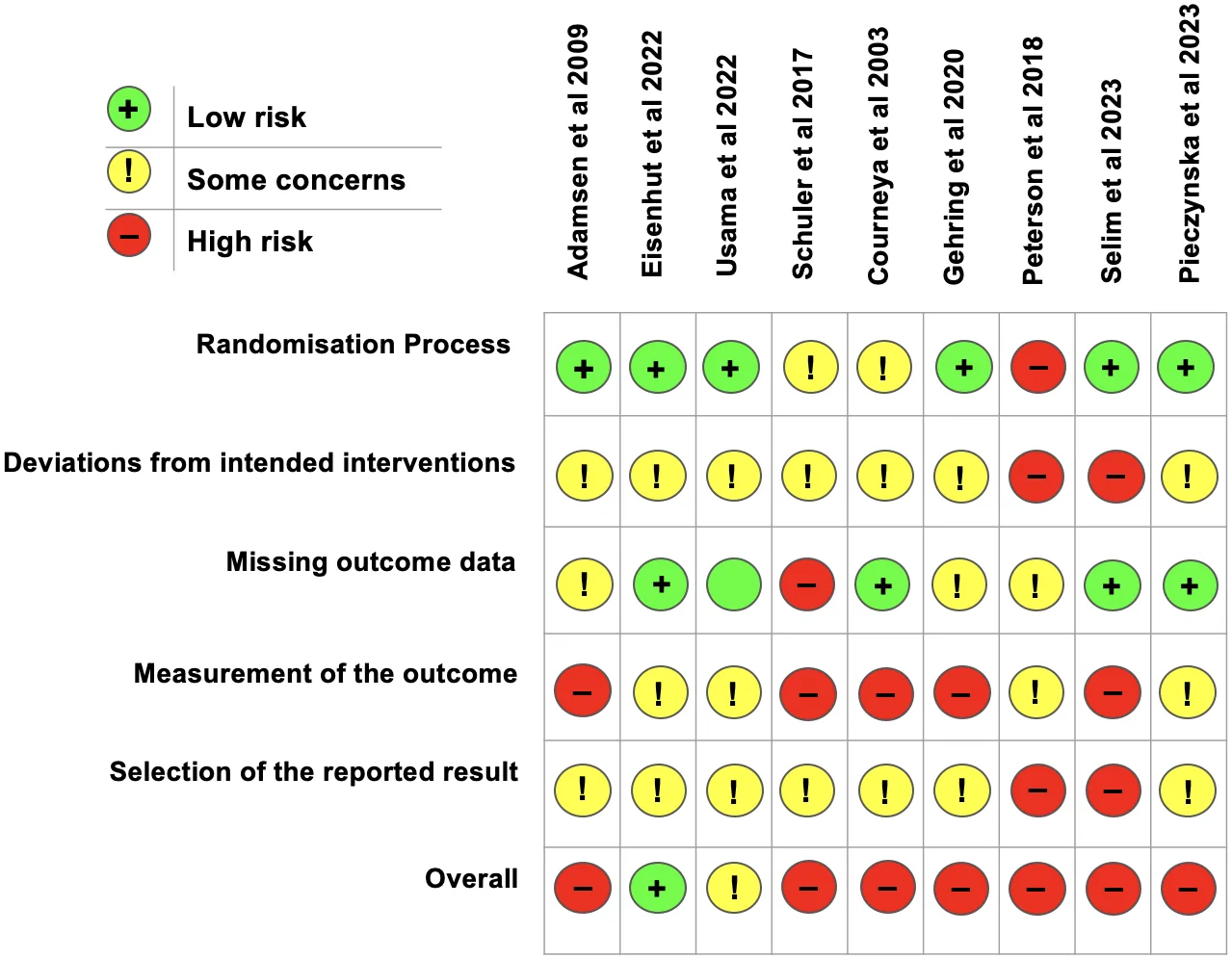
Summary of findings of cochrane risk of bias 2 assessment for randomized studies.

In total, we identified 4210 articles from two different data bases, as shown in **Figure 1**. After two-step screening, 3052 articles were excluded based on title while 1107 articles were excluded after reviewing the abstract as they were considered not to be relevant to our research question. A full text review of the remaining 51 articles was conducted and 20 studies were excluded due to strictly addressing rehabilitative intervention in 5 studies, 5 studies that only had listed protocols with no results at the time of writing this manuscript, 3 studies that only had behavioral interventions such as cognitive exercises and meditation, 2 pre-clinical studies and 5 articles that did not include brain tumor patients. A total of 31 studies were included in this systematic review including 23 clinical trials (9 randomized controlled trials and 14 other clinical trials including prospective single arm feasibility study, pilot feasibility study, randomized cross-over study and explorative interventions), 4 observational studies, 2 qualitative analyses, 1 case report and 1 case study (**Table 1**). The randomized controlled trials were classified as level 1b evidence according to the Oxford CEBM Levels of Evidence guidelines (16). Using the Cochrane Risk of Bias 2 (RoB 2) tool, most trials were judged to have an overall high risk of bias, **Figure 2**. Two exceptions were identified - the study by *Eisenhut* et al. (2022), which was consistent with a low risk of bias, and the study by *Usama* et al. (2023), which revealed only some concerns regarding deviations from the intended intervention and outcome measurement, primarily due to self-reported outcomes and lack of participant blinding (18, 19).

**Table 1:** Exercise intervention studies meeting criteria for review.

Study	Publication year	Study Design	Patient Population and Brain Tumor Type	Cancer-directed Treatment Interventions	Sample size	FITT (Frequency, Intensity, Time duration)	Type of Exercise	Control group	Total duration	Outcome measures	Main findings	CEBM level of evidence	Intervention related Adverse events	Limitations
Jost-Engl et al	2025	Single- arm clinical trial	Patients with newly diagnosed high grade glioma undergoing chemotherapy	Surgery + radiochemotherapy	54	Endurance training: F- 2 times/ week’ I- moderate (70% HR) T-50 mins /session Strength training: F- 2 times/ week’ I-moderate to high (17-20RPE) T- 50 mins/session	Supervised individualized in-person sessions including endurance and strength training.	none	16 weeks	Primary end-point: cardiorespiratory fitness (PWC). Secondary endpoint: QoL, Peak oxygen uptake (VO2) and peak power output (Ppeak)	(PWC), (VO2 peak) and (Ppeak) improved significantly with comparatively larger gain in the first 8 weeks. QoL improved in the first 8 weeks and remained stable through week 16.	2b	Grade 1–2 exercise related AE’s reported according to the CTCAE severity scale. These included dizziness, buttock pain, sinus bradycardia, localized edema, chest pain, vomiting and bronchial obstruction.	Single-arm design, natural recovery processes may have contributed to the observed improvements, higher drop-out rate, in-person supervision limits broader clinical implementation.
Avancini et al.	2024	Prospective single-arm feasibility study	Different kinds of tumors including 3 brain metastasis patients (mean age 60.5) (56.8% male & 43.2% female)	Not specified	44	Aerobic: F- 2 times per week I- moderate (3-5/10 RPE) T- ~30 min combined exercise Resistance: F- 2 times per week I-moderate (3–5 RPE) T- ~30 min combined exercise/session	Home based, personal training and group based, aerobic and resistance training	none	3 months	Primary: Recruitment and completion rate, attendance, adherence, patient safety Secondary: Cardiorespiratory fitness, muscle strength, anthropometric measures, QoL.	Median attendance rate 92%, adherence rate 88%, improved cardiopulmonary fitness. Improved physical, emotional, functioning, fatigue domains of QoL	4	Dizziness, Back pain, Leg pain, Palpitations.	Single-arm design
Kohler et al	2024	Qualitative analysis	Parents and patients of post fossa brain tumor (mean age 10.6)(83% male)	Not reported	11	F- 1×/week supervised + 3×/week home I- Moderate–vigorous (HR ≥140 bpm) T- 30–60 mins/session	Face-to-face delivered Goal-directed therapeutic exercise.	none	12 weeks	Perceived improvements, program logistics, connections with therapist, activity selection, technology options.	Perceived Improvement in movement competence	Not applicable	None reported	Qualitative analysis
Selim et al	2023	RCT	Pediatric medulloblastoma patients with ataxia after resection(age 5-10yrs)	surgery	30	F- 3 times/week I- NA T- 60 mins/session	Cognitive task + balancing task + chosen physical therapy (dual group)	Chosen physical therapy	8 weeks	Stability test (HUMAC balance system), pediatric balance scale, functional independent measurement	Improvement on the pediatric balance scale and stability test in the dual group. No difference in the functional independent measurement between two groups.	1b	None reported	The negative effect of therapy related fatigue could not be eliminated. Longer training time in the intervention group could have affected results.
Pieczynska et al	2023	RCT	Adult patients with stage III/IV glioma (mean age 52.58)	Surgery + RT	72	F- 5 times/week I- Moderate (~70% HRmax) T- 60 mins/session	At-hospital supervised physical exercise along with RT followed by at home physical exercise	Normal activities with minimum level of physical activity	1 month	Hand grip test, 6-minute walk test, time up and go test, functional independence measure, QoL, fatigue, anxiety and depression and cognition.	No significant difference in 6MWT, FIM, QoL, fatigue, anxiety and depression. Decreased HGS and cognition domain in control.	1b	None reported	Self-reported level of physical activity in the control group, interaction between studied variables not studied.
Nowak et al.	2023	Pilot feasibility study	Patients with High-grade g lioblastoma scheduled to receive chemoRT (mean age 51.03)	Surgery + adjuvant chemo-RT	25	Aerobic: F- 2 times/week I-Moderate–vigorous (60–85% HRmax) T- 60 mins/session Resistance: F- 2 times /week I- moderate to vigorous (60-85% of 1RM) T- 60 mins/session	Supervised aerobic + resistance exercise	none	Variable (Duration of their chemoradiotherapy treatment)	Primary: feasibility Secondary: DEXA scan, fatigue, physical function tests, QoL, sleep quality	Low-to-high feasibility (Adherence= 90%, compliance=43% with 17 participants completing the study) and Significant improvement in lower limb strength tests. Not significant for other outcomes	4	None reported	Not controlled, small sample size, selection bias
Sandler et al.	2023	Feasibility study	Primary Brain tumor patients 12–26 weeks post-radiotherapy (mean age 51)	Radiotherapy	12	Aerobic: F- 2 times/ week I- moderate T- 150 mins Resistance: F- 2 times/week I- 600 METs/min T- 150 mins	>150 mins of weekly moderate intensity exercise (aerobic +resistance)	none	18 weeks	Primary: feasibility Secondary: QoL, fatigue, physical function, aerobic fitness, hospital anxiety and depression,	Study was feasible (recruitment 80% and retention 92%) with improved QoL, physical activity, aerobic fitness, depression and lower body strength.	4	Fall, dizziness, skin abrasion, headache, lower back pain, fatigue, unilateral knee pain.	Single-arm design
Dulger et al.	2022	Randomized cross-over study	Female patients with pituitary macroadenoma post-resection (age 18–65 yrs)	Surgery	10	Aerobic: F- 3 times/week I- Moderate (<50–70% HRmax) T- 30 mins/session Strength training: F- 3 times/week I- <50% of 1RM T- 30 mins/session Yoga: T- 60 mins/session	aerobic +strength (A+ST) training vs yoga	None	6 weeks	QoL, fatigue, sleep quality, sexual function, cognitive function, emotional state	Significantly improved QoL and cognition in the yoga group and significantly improved cognition and anxiety in the A+ST group	1b	None reported	Small sample size
Eisenhut et al	2022	RCT	Patients with high grade glioma after surgery and during chemoradiotherapy (age 18–75 yrs)	Surgery+ chemoradiotherapy	29	Endurance training: F- 2 times/week I- 11–14 Borg scale T- 35–45 mins/session Strength training: F- 2 times/week I- 11–15 Borg scale T-35–45 mins/session	Endurance training (ET)vs strength training (ST)	No physical activity	6 weeks	Depression, anxiety, perceived stress, insomnia, fatigue, physical fitness,	HGS, insomnia and perceived stress improved more in the ET and control group vs ST group. Fatigue increased in the ET and ST and decreased in the control group	1b	None reported	Small sample size
Usama et al.	2022	RCT	Children with posterior fossa tumor (age 5–12 yrs)	Not specified	60	F- NR I- NA T- 60 (control intervention)+30 (balance OR coordination)mins/session	Control intervention (CI)+ Balance exercise (HUMAC balance and tilt system) vs CI+coordination exercise	Pilates Core stability exercise	12 week	Change in balance (postural stability scores and coordination scores)	Significant difference between balance and control groups Significant difference between coordination and control group in coordination scores only.	1b	None reported	None specified
Spencer et al	2021	Pilot feasibility study	Patients with high grade glioma	Radiochemotherapy	19	F- once weekly education session + twice weekly strength training with 150 mins per week of aerobic exercise I- NR T- 60 mins (education sessions)	Cardiorespiratory exercise and strength training in-person (exercise group) or just education (education group)	Usual care group	10 week	Feasibility, retention, QoL, fatigue, cardiorespiratory fitness (6MWT), flexibility and strength	High feasibility (63% accrual, 80% adherence, 2/19 attrition rate )Less fatigue, improved QoL and favorable changes in the cardiorespiratory fitness, flexibility and strength in the exercise group compared to education and control group.	4	None reported	Limited generalizability, small sample size, shorter duration and restricted recruitment due to COVID-19.
Cox et al.	2020	Controlled crossover trial	Pediatric hemispheric or posterior fossa brain tumor survivors (6–17 years)	radiotherapy	32	F- 3 times a week for group and 4 times for combined settings I- moderate to vigorous (80% of HR) T- 90–120 minutes	Aerobic exercise either in group or combined group/home setting.	No exercise/ normal physical activity	12 week	Behavior (Go task and go/no-go task) and functional connectivity	Significant increase in response during the go/no-go task exercise group. No significant change in response latency and resting state connectivity.	1b	None reported	Small sample size, heterogeneity in patient characteristics
Gehring et al	2020	Pilot RCT	Patients with stable grade II or stable grade III glioma (age	Not specified	34	F- 3 times /week I- moderate to vigorous (60-85% HR) T- 20–45 mins/ session	Home based remotely coached aerobic exercise group	Active lifestyle according to the Dutch public health guidelines	6 months	Cognitive performance, fatigue, sleep, mood, QoL	Improved sleep, fatigue, mood, mental health related QoL, verbal memory, executive dysfunction and several attention measures in the exercise group. No differences in tumor specific QoL. improved sustained selective attention in the control group.	2b	None reported	None specified
Halkett et al.	2020	Qualitative study	Glioblastoma patients scheduled to receive chemoradiotherapy (age >18 yrs)	Surgery + chemoradiotherapy	19 patients + 15 carers	Aerobic: F- 3 times/week I- moderate to vigorous (60-85% HR) T- 60 mins /session Resistance: F- 3 times/week I- moderate to vigorous T- 60 mins/session	Supervised exercise therapy (aerobic+resistance)	None	7 weeks	Patients perspective in participation and perceived outcome including physical and mental well-being	Expressed positive perceptions and experiences including improvements in health, regaining a sense of control, keeping active and benefits of carers.	Not applicable	None reported	Patients who did not participate could not be interviewed.
Troschel et al.	2020	Pilot study	Brain tumor patients (mean age 47)	Surgery + chemoradiotherapy	9 patients + 6 relatives	F- daily I- NR T- 2–4 hrs/session	Ski-based exercise	none	1 week	Feasibility, safety, fitness, QoL, anxiety, depression, distress and adverse events	Feasibility (recruitment:44/170 interested, attrition: 15 withdrew, 5 excluded),Increased QoL and decreased distress after intervention	4	None reported	Did not employ detailed statistical analysis, small sample size.
Troschel et al.	2019	Case report	33 yr old male with glioblastoma multiforme	Surgery + chemoradiotherapy	1	F- variable - vigorous (70-75% of HR) T- variable	High intensity training like Running, cycling, walking. power training, soccer, swimming, skiing	none	Throughout the treatment of the patient (around 2 years)	Feasibility, adherence, QoL and adverse events	High-intensity, long term exercise is feasible with good adherence. Improved mood, well-being and QoL reported	4	None reported	Case report
Levin et al	2019	Case study	Depressed brain tumor survivors; Anaplastic oligodendroglioma and glioblastoma multiforme (age 58 and 61)	Surgery + chemoradiotherapy	2	Aerobic: F- 2 times /week I- moderate to vigorous T- 20 mins/session Resistance: F- 2 times/week I- moderate to high T- 40 mins/session	Supervised Moderate to vigorous aerobic exercise and vigorous training.	none	12 weeks	Aerobic adaptations (VO2 and HR), muscle strength (chest press, leg press), QoL, sleep quality, mental health outcome (depression, anxiety, total distress), cancer specific self-efficacy, adherence	Improved cardiovascular efficiency, maximal strength increased by 17% and 48%, improved mental health outcome. 100% adherence	4	None reported	None specified
Colledge et al	2018	Exploratory intervention study	Post-aneurysmal SAH and meningioma patients and healthy controls (mean age 58.5)	Surgery only	48	F- NR I-NR T-NR	Individualized exercise program with a standard energy expenditure of 17.5 kcal/kg/week	Healthy patients as control	12 weeks	Psychological functioning, verbal learning and memory, sleep quality	Decreased depression and insomnia in aSAH and meningioma patients, decreased perceived stress in meningioma and increased in aSAH patients. Increased total learning performance in all groups.	2b	None reported	Interpretation carried on descriptive statistics with no power calculation, selection bias, no control group of non-exerciser.
Lerch et al	2018	Controlled cross-over clinical trial	Pediatric brain tumor survivors (age 6–17 yrs)	Radiotherapy +- chemotherapy	28	F- 3–4 times/week I-vigorous (80% of HR) T- 30–90 mins /session	Aerobic exercise	No exercise	12 weeks	Cortical thickness, regional cortical thickness, relation between behavior and regional cortical thickness.	Increased cortical thickness in right pre and post-central gyri, left pre and post-central gyri, left temporal pole, left superior temporal gyrus, left parahippocampal gyrus.	1b	None reported	No observation of carry-over/maintenance affects over 12 weeks
Peterson et al.	2018	Randomized Pilot study	Cancer survivors (mean age 57.9) including 1 brain metastasis (NSCLC)and 1 primary brain tumor (anaplastic oligodendroglioma)	None specified	28 (2 brain tumor patients)	F-NR I- aerobic: moderate (55–65% HR) T-30 mins/session	Aerobic training (AER) or cognitive training (COG) or AET+COG	Flexibility only intervention	12 weeks	Physiological assessment (vitals, anthropometry, strength, cardiovascular fitness) and cognitive (memory, executive function, attention, reasoning)	Memory improved in the AER group and verbal fluidity increased in the control group. No increase in cognitive assessment in the COG or COG+AER group	2b	None reported	Complexities of cancer
Milbury et al.	2017	Single arm pilot trial	Patients above 18 yrs with high grade glioma having completed at least 4 weeks of radiotherapy	Surgery + chemoradiotherapy	5 dyads (patient + care-giver)	F- 2–3 times/week I- NR T- 60 mins/session	yoga	None	12 weeks	Self-reported symptoms, QoL, depressive symptom, sleep disturbances, fatigue	Decreased cancer-specific symptoms, sleep disturbances and improved QoL.	4	None reported	No control group and small sample size
Piscione et al.	2017	Controlled cross-over clinical trial	Patients with age 6–17 with hemispheric or posterior fossa tumor treated with radiation	surgery+radiation	32	F- 3 per week in group and 4 per week in combined I- vigorous (80% HR) T- 90 mins /session (group) and 30 + 90 mins/session (combined)	Aerobic training	No exercise	12 weeks	Physical function (motor performance, coordination, balance, strength) and fitness (peak oxygen consumption)	Improvement in bilateral co-ordination with below average assessment in motor performance.	1b	None reported	Small sample size, un-blinded assessors
Reed et al.	2017	cross -sectional Feasibility study	High-grade glioma patients aged 18 yrs and older.	Surgery + chemoradiotherapy	16	F- NR I-NR T-NR	Cycling exercise	None	–	Fitness (grip strength, resting and peak heart rate and B.P, VO2 and leg fatigue), physical activity levels, QoL	Clinically significant decrease in QoL, total PA levels correlated positively with QoL measures	4	None reported	Small sample size, cross-sectional
Schuler et al.	2017	RCT	Patients with Different types of cancer including 6 brain tumor patients.	Not specified	70 (6 brain tumor patients)	F- 3 times/week endurance and 2 times per week strength training I- moderate (borg>13) T- 20–30 mins/session	Structured individual exercise program+-physical therapy	No physical exercise	12 weeks	Fatigue (general, mental and severe), walking distance, blood parameters (albumin)	No significant difference in general fatigue, decrease in mental fatigue in exercise group only. At 12 weeks, the exercise+PT group showed an increase in albumin levels.	1b	None reported	Not blinded so confounding factors including educative influence leading to increased metabolic equivalents in control group.
Baima et al	2016	Single arm prospective trial	Brain tumor patients aged 18 or older (high grade astrocytoma and brain metastasis)	surgery	15	F- daily I- NR T- 20 mins /session	Resistance band exercises, balance exercise and walking recommendations.	none	1 month	Compliance, safety, QoL	64% continued exercise on a regular basis, increase in brain cancer specific well-being and QoL.	4	Dizziness, muscle soreness,	Small sample size, large heterogeneity in tumor biology, small sample size,
Grabenbaeur et al.	2016	Controlled prospective feasibility trial	Patients with cancer on or after treatment with age>18 yrs. Includes 3 brain tumor patients	chemoradiotherapy	45 (3 brain tumor patients)	F- 3 times/week I- NR T- 30–60 mins / session	Individually tailored exercise with increased intensity	None	12 months	Adherence, Cardiopulmonary fitness (VO2, heart rate, max power), body composition, QoL.	Adherence for 3,6 and 12 months was 93%, 91% and 82%. Decrease in median BMI and total fat mass, median peak O2 consumption increased, significantly improved QoL.	2b	Progression in lymphoma of one patient and anal discomfort in a patient with history of anal cancer.	None specified
Williams et al.	2014	Prospective cohort study	Adult runners and walkers with brain tumors.	Not specified	110	FITT does not apply	Running and walking, measured in terms of energy expenditure in METs.	none	–	Relation of brain cancer deaths with MET hours per day run or walked (Cox proportional hazard analysis)	Brain cancer mortality was 43.2% lowered for 1.8 to 3.5 MET hr/d exercise and 39.8% lowered for those who exercised ≥3.6 MET hr/d.	2b	None reported	None specified
Ruden et al.	2011	Prospective cohort study	Patients older than 18 with grade3 or 4 recurrent glioma.	Surgery + chemoradiotherapy	243	FITT does not apply	Mild (3), moderate(5) and strenuous (9) intensity exercise expressed as average MET hrs/week.	None	27.43 months follow up	Exercise behavior, functional capacity and effect on survival (Cox proportional hazard analysis)	Functional capacity is an independent predictor of survival, median survival was 13.03 months and 21.84 months for exercise < 9MET hr/d and ≥9 MET hr/d respectively.	2b	Not reported	Results only generalizable to patients with KPS ≥ 70,
Adamsen et al.	2009	RCT	Patients with cancer age 18–65 years and had at least 1 cycle of chemotherapy	chemotherapy	269 (5 brain tumor patients)	F- 4 times / week I- physical training: high intensity T- physical+relaxation training (90 + 30) mins /session, body awareness +relaxation (90 + 30) mins/session and 30 mins massage sessions	High intensity cardiovascular and resistance training, relaxation and body awareness training, massage	Conventional care + allowed freely to increase activity	6 weeks	Primary outcome: Fatigue, QoL and physical activity Secondary outcome:muscle strength, aerobic capacity	Improved fatigue and physical capacity including maximum oxygen capacity and strength. No significant effect on QoL.	1b	One case of seizure after training, a few cases of leucopenia and increased heart rate/B.P.	Could only report immediate effect, personnel delivering exercise sessions collected data (potential bias), all patients had good performance status.
Courneya et al.	2003	RCT	Patients with cancer (mean age 51.55). Includes 3.1% Brain tumor patients	Not specified	108 (3.1% brain tumor patients)	F- 3–5 times/week I- moderate (65-75%) T- 20–30 mins /session	Group psychotherapy (GP) and home-based personalized exercise program (EX)	None	10 weeks	QoL, satisfaction with life, depression, anxiety, fatigue, physical fitness, cardiovascular endurance, flexibility of the lower back.	Improved QoL, physical fitness, flexibility, cardiovascular endurance in the GP+EX group. Decreased fatigue with borderline improvement in satisfaction with life in the GP+EX group compared to GP alone.	1b	None reported	Full factorial design was not used, unsupervised (based on self-reported measures of exercise), no long term follow-up, may not be generalized, adherence and contamination problems.
Jones et al.	2009	Cross-sectional observational study	Patients with recurrent grade 3 or 4 malignant glioma (age >18 yrs)	Not specified	171	FITT does not apply	Self-reported exercise behavior	none	–	Functional capacity (6MWT), QoL, performance status.	Performance score (KPS), self-reported exercise and QoL increased across 6MW disease quartiles. 6MW test and KPS are significantly correlated (r=0.34), with QoL as well (-0.43 to +0.46)	4	None reported	Selection bias (low eligibility rate), small sample size, cross-sectional study design.

HR, heart rate; NR, not reported; RPE, rate of perceived exertion; VO_2_, volume of oxygen; PWC, physical working capacity; CTCAE, common terminology criteria for adverse events; RM, repetition maximum.

All nine studies reported the use of randomization; however, unclear or inadequately described allocation concealment raised concerns about potential selection bias in two studies (20, 21) and allocation concealment was explicitly compromised in one study due to reassignment of five participants (22).

Concerns regarding selective reporting were present across most studies, with the exception of two trials that did not specify primary or secondary outcomes (22, 23). Additionally, missing outcome data resulting from death, participant refusal, physical impairment, treatment-related side effects, and medical events contributed to further risk of bias in outcome assessment.

**Figure 3:**
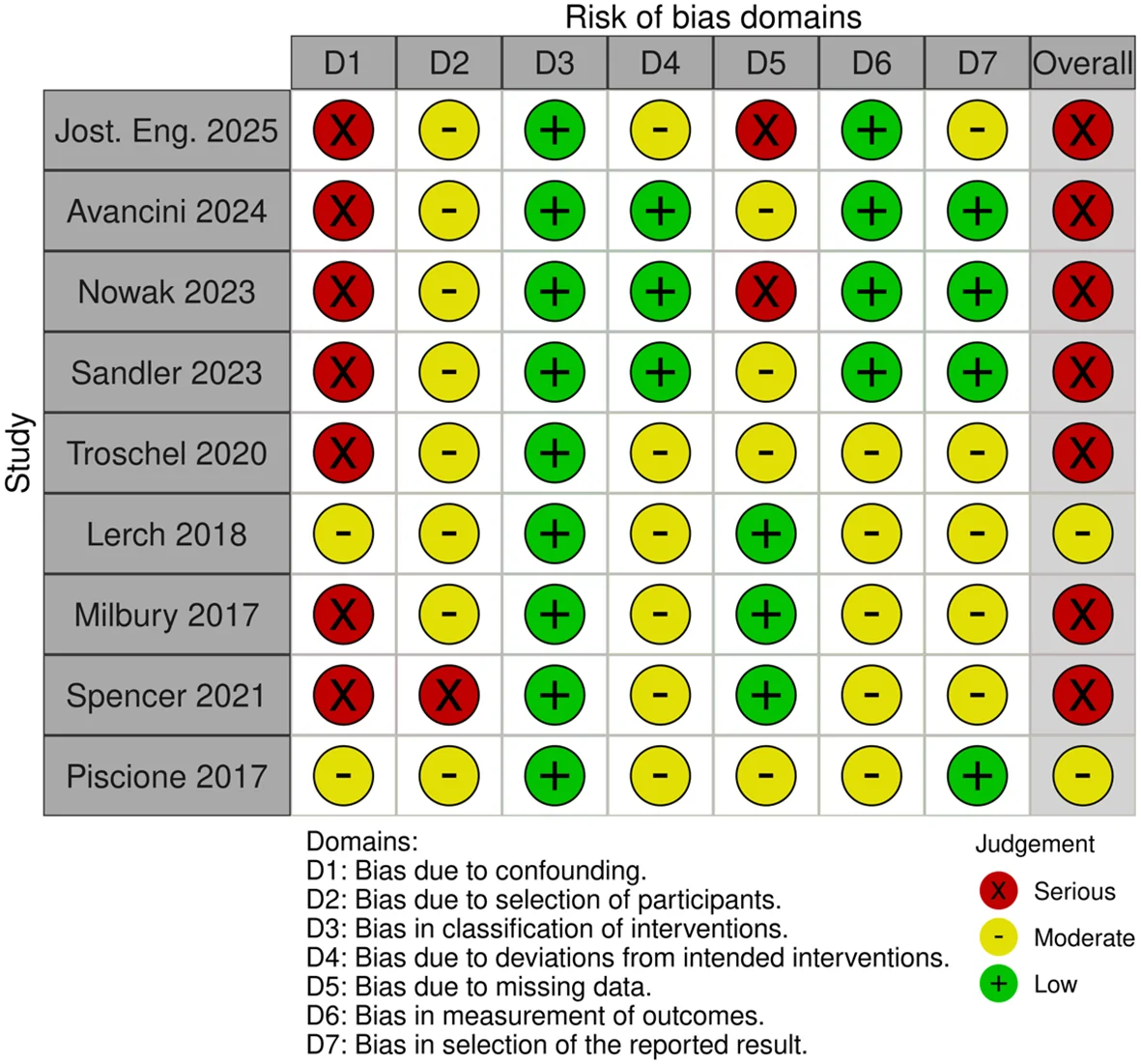
Summary of findings of risk of bias in non-randomized studies of interventions (ROBINS-I) assessment for non-randomized studies.

For the non-randomized studies amenable to assessment with the Risk of Bias in Non-randomized Studies of Interventions (ROBINS-I) tool, most studies were judged to have an overall serious risk of bias, with the exception of Piscione et al., 2017 and Lerch et al., 2018 which had a moderate risk of bias, **Figure 3**. Overall, all the studies had a moderate to high risk of bias in confounding with a low to moderate risk of bias due to classification of interventions, deviations from intended interventions, measurement of outcomes and selection of reported results.

Other studies ranged from level 2b (controlled cross-over clinical trials) to level 4 (feasibility and cross-sectional studies) according to the Oxford CEBM Levels of Evidence guidelines (16) Across the included studies, a total of 1114 patients with brain tumors (primary: 1110; brain metastases: 4) were identified. Of these, 1092 patients were derived from brain tumor–specific studies and 22 from mixed cancer cohorts reporting the number of brain tumor participants. Overall, 198 patients were allocated to intervention groups and 149 to control groups, while 767 patients were included in single-arm or non-comparative study designs without a control group.

The physical exercise interventions included exclusive aerobic exercise in 10 studies, individualized exercise programs or self-reported patient initiated exercise programs in 6 studies, balance and coordination exercises in 2 studies, yoga in 1 study and 12 studies with mixed interventions such as aerobic exercise and strength training. See **Table 1** for a detailed description of the interventions evaluated in each study. Given the heterogeneity across studies, findings are presented descriptively. Data were extracted into a structured table and summarized at the individual study level. Studies were grouped according to exercise type, and findings were reported narratively within each group without statistical pooling or formal cross-study comparison (**Table 1**).

### 3.1 Aerobic exercise interventions

Nine studies evaluated aerobic exercise interventions, including three crossover trials, two pilot studies, one single-arm study, two observational studies, and two case reports. *Cox* et al. (2020) conducted a controlled cross-over clinical trial in pediatric patients with hemispheric or posterior fossa brain tumors. In this study, patients completing a 12-week moderate-vigorous (Heart rate–HR > 80%) aerobic exercise program performed in 30–90 minutes sessions 3–4 times a week, demonstrated improvement in behavior in the form of a go/no-go task performance while other aspects of behavior and functional connectivity such as response latency and resting state connectivity showed no significant change (24). Another controlled crossover clinical trial in pediatric patients compared the cortical thickness between patients doing vigorous aerobic exercise 3–4 times per week in 30–90 minutes sessions versus no exercise and the relation between cortical thickness and behavior (25). The results showed increased cortical thickness in pre and post-central gyri and left temporal and parahippocampal gyri in the exercise group after 12 weeks (25).

*Piscione* et al. (2017), in their controlled crossover clinical trial reported improvement in physical function in the form of bilateral coordination while below average motor performance in pediatric patients with hemispheric or posterior fossa tumors undergoing vigorous (80% HR) aerobic training 3 to 4 times per week in 30–90 minutes sessions compared to control (26). A randomized pilot study conducted in cancer survivors included a patient with metastatic brain tumor and another with anaplastic oligodendroglioma. They studied aerobic and cognitive training individually as well as combined and reported improvement in memory in the aerobic group (performing moderate aerobic exercise in 30 minutes sessions) with no overall improvement in cognition (22).

*Troschel* et al. (2020), in their pilot study, measured the effectiveness of daily ski-based exercise (2–4 hours) in adult patients with brain tumors and their relatives for one week. The feasibility was assessed via recruitment metrics. Of 170 individuals contacted, 44 (26%) expressed interest; 15 withdrew and 5 were excluded, resulting in 15 participants. The study reported increased QoL and decreased emotional distress (27). In a single-arm prospective clinical trial, adult patients with high-grade astrocytoma or brain metastases performed resistance and balance exercises daily in 20-minute sessions and were provided walking recommendations for one month. Although only 64% adhered to the exercise intervention, the overall study showed improved well-being and QoL (28).

In a cross-sectional study conducted in adult high-grade glioma patients, cycling exercise showed clinically significant decrease in QoL but there was a positive correlation between total physical activity and QoL both at baseline and after intervention (29). *Williams* et al. (2014) conducted a prospective cohort study in which adult runners and walkers with brain tumors were studied for the impact of their lifestyle on the mortality rate. The intervention was measured in terms of energy expenditure reported as Metabolic Equivalent of Task (METs) hours/day and were correlated with mortality which was reported to be lowered by 43.2% with the MET rate ranging from 1.8-3.5 hours/day and lowered by 39.8% in those who exercised more than or equal to 3.6 MET hours/day (30).

A 33-year-old male with glioblastoma was followed throughout the course of the disease and treatment. He actively performed high-intensity exercise such as running, cycling, power training, soccer, swimming and skiing for 2 years showing that this high-intensity exercise is feasible and the patient reported improved mood, well-being and QoL. The patient stopped exercising 6 weeks before his death (31). In a case series, 2 patients were included, one with anaplastic oligodendroglioma and another with glioblastoma. They performed supervised moderate-to-vigorous exercise 2 times per week in 60-minute sessions for 12 weeks, the intervention resulted in improved cardiovascular efficiency with a 17% increase in strength and 48% improvement in mental health (32).

Overall, aerobic exercise interventions in brain tumor patients were feasible and generally safe, with some studies showing improvements in physical fitness, quality of life, or cognitive outcomes, while others reported minimal or mixed effects, and adherence varied across studies. It must be noted that the small numbers of brain tumor patients, particularly in mixed-cancer cohorts, limits the generalizability of the findings in most of these studies.

### 3.2 Mixed exercise interventions

Several studies evaluated combined aerobic and resistance training interventions in brain tumor patients. These included four randomized controlled trials, one randomized crossover trial, one cohort study, four feasibility studies, and two qualitative studies.

In their randomized controlled trial, *Pieczynska* et al. (2023) studied adult patients with high-grade glioma completing supervised moderate intensity physical exercise followed by home based exercise performed 5 times per week in 60-minute sessions along with radiotherapy for one month. The authors did not detect differences in the 6-minute walking test (6MWT), functional independence measures, QoL or fatigue, but decreased cognition was reported in the control group that performed a minimal level of physical activities (33).

Another randomized controlled trial in patients with high-grade glioma, evaluated 6 weeks of endurance training versus twice weekly strength training in 35-45-minute sessions, compared with sedentary healthy controls. The authors report decreased stress and insomnia, as well as improved hand grip strength in the endurance group compared to the other two groups while fatigue was reported to be increased in both endurance and strength training groups compared to control (18). *Adamsen* et al. (2009) evaluated an intervention consisting of high-intensity cardiovascular and resistance training, body awareness relaxation training and massage for 2 times per week in 120-minute (90 minutes training + 30 minutes relaxation) sessions for 6 weeks in adult cancer patients including five brain tumor patients, comparing them to control patients that were allowed to freely increase their level of physical activity. The results showed improved fatigue, physical ability and strength among patients in the intervention group with no improvement in QoL (34).

*Gehring* et al. (2020) conducted a pilot randomized controlled trial in patients with grade 2/3 gliomas. The patients performed moderate to vigorous home-based supervised exercise 3 times per week in 20-45-minute sessions and were compared with patients with an active lifestyle for 6 months. The results showed improved fatigue, mental health, sleep, mood, verbal memory, attention, executive function and overall QoL, with no difference in tumor-specific QoL (35).

In their randomized crossover study in post-resection female patients with pituitary macroadenoma, *Dulger* et al. (2022) reported significantly improved anxiety in the group performing moderate intensity (HR <50-70%) aerobic exercise combined with moderate intensity strength training (<50% of 1 Repetition Maximum- RM) compared to another group performing yoga for 3 times a week in 30 minute sessions, although significantly improved QoL in the yoga group was noted (36).

In a large prospective cohort study including 243 adult patients with recurrent high-grade glioma the level of exercise activity was characterized based on average MET hours/day divided as mild (3 MET hour/day), moderate (5 MET hour/day) and strenuous (9 MET hours/day). The patients were followed along the course of their treatment and the results reported a median survival of 13.03 months for patients exercising less than 9 MET hours/day and 21.84 months for those exercising more than 9 MET hours/day (37).

In a prospective single-arm feasibility study, including cancer patients including 3 brain metastasis patients, moderate intensity (rating of perceived exertion–RPE 3-5) home-based personal training and group-based aerobic and resistance training performed 2 times a week in 30-minute sessions was studied. The median attendance rate was 92% with 88% adherence and overall improved cardiopulmonary fitness, physical and emotional functioning, as well as improved fatigue (38).

Another feasibility study assessed adherence, compliance and retention in adult patients with glioma performing supervised moderate-vigorous intensity (60-85% HR) aerobic and resistance exercise (60-85% 1RM) 2 times per week in 60-minute sessions along with their chemoradiotherapy treatment (39). Among participants who completed the intervention, mean adherence was high at 90% (range 33–100%), while compliance was lower, averaging 43% (range 24–83%). A total of 17 participants completed the intervention and post-intervention assessments. Significant improvement in lower limb strength without significant findings in Dual-Energy x-ray Absorptiometry (DEXA) scan, fatigue, physical functioning, QoL and sleep quality was reported (39). *Sandler* et al. (2024) demonstrated overall feasibility of their protocol assessed by recruitment (80%) and retention (92%) with improved QoL, physical activity, aerobic fitness, depression and lower body strength in adult primary brain tumor patients post-radiotherapy who performed at least 150 minutes of moderate intensity aerobic and 600 MET-minutes of resistance exercise weekly for 18 weeks (40).

High-grade glioma patients performing moderate to high intensity aerobic and strength training exercise 3 to 5 times a week in 10-30-minute sessions were compared to usual care or exercise education for 10 weeks in another feasibility study (41). Feasibility was assessed via accrual, attrition, and adherence/attendance. Of 30 patients screened, 19 (63%) were enrolled and 17 completed the study. Mean attendance was 80%, and 90% of participants completed weekly logs. The results showed improved QoL, fatigue and improved cardiopulmonary fitness, strength, flexibility in the exercise group compared to the usual care group and the exercise education group (41).

Lastly, we also reviewed two qualitative analyses, in which perceived outcomes were reported by investigators. *Kohler* et al. (2024) reported an improvement in movement competence in pediatric posterior fossa brain tumor patients performing in-person, vigorous (HR >140 bpm) once a week supervised then 3 times a week at home in 30 to 60-minute sessions, goal-directed therapeutic exercises (42). *Halket* et al. (2021) on the other hand, studied supervised exercise therapy including moderate to vigorous aerobic resistance training 3 times a week in 60-minute sessions, in adult glioma patients scheduled to receive chemoradiotherapy. Caregivers were also invited to share their perception of the efficacy of the intervention. The patients and caregivers evaluated the intervention positively and reported subjective improvement in health and regaining a sense of control (43).

Overall, combined aerobic and resistance training was generally feasible and safe, with adherence ranging from moderate to high. Improvements were reported in physical fitness, strength, fatigue, and some aspects of QoL. Cognitive outcomes were inconsistent, with occasional improvements in memory or anxiety, while other domains showed minimal change. Some studies reported no significant differences in functional independence, fatigue, QoL, or body composition. Outcomes varied across studies, with small numbers of brain tumor patients in mixed cohorts limiting generalizability. As noted before, the small number of brain tumor patients limits generalizability *(*38*).*

### 3.3 Balance and coordination interventions

Two randomized controlled trials evaluated balance and coordination exercise interventions in pediatric brain tumor patients. *Selim* et al. (2023) conducted a randomized clinical trial in which pediatric patients with ataxia after medulloblastoma resection performed balance exercises 3 times per week in 60-minute sessions alongside cognitive tasks and physical therapy (23). The control intervention was physical therapy only with results showing improvement in the pediatric balance scale and stability test with no difference in functional independent measurement between the intervention and the control group (23).

In another randomized controlled trial, pediatric patients with posterior fossa tumors performed either pilates based stability exercises only (control group) in 30-minute sessions or pilates along with balance exercise (balance group) with additional 60-minute sessions or with coordination exercise (coordination group). The results showed significant difference in balance and coordination scores between balance and control group while only significant difference in coordination scores between coordination and control group (19).

Overall, balance and coordination interventions were feasible and associated with improvements in balance and coordination outcomes. Improvements were observed in balance and coordination scores compared with control groups. Functional independence measures did not differ between intervention and control groups. Outcomes varied depending on the type of exercise and specific measures assessed.

### 3.4 Yoga

A single arm pilot trial enrolled adult patients with high-grade glioma having completed at least four weeks of radiotherapy along with their caregivers to study the effectiveness of yoga on QoL, depression, fatigue and sleep disturbances. After twelve weeks of yoga sessions lasting 60 minutes performed 2 to 3 times per week the patients reported decreased cancer specific symptoms, such as sleep disturbances and improved QoL (44).

### 3.5 Individualized and self-reported exercise programs

Six studies evaluated individualized or self-reported exercise approaches rather than a predefined exercise intervention. These included two randomized controlled trials, one single-arm clinical trial, one feasibility study, one exploratory intervention study, and one cross-sectional observational study. In the randomized controlled trial, cancer patients including 6 brain tumor patients were included and outcomes including fatigue, walking and serum albumin levels were compared between patients performing a moderate intensity (Borg >13) structured exercise program with or without physical therapy 2–3 times a week in 20-30-minute sessions and patients performing no exercise (21). Although the results showed no significant difference in general fatigue, a decrease in mental fatigue and increased albumin levels in the group performing both exercise and physical therapy was reported. *Courneya* et al. (2003) also studied different types of cancer in their randomized controlled trial, out of which 3.1% were brain tumor patients. The study reported improved QoL, physical fitness, flexibility, cardiovascular endurance in the group performing both moderate intensity (HR 65-75%) exercise and group psychotherapy for 3–5 times a week in 20-30-minute sessions compared to psychotherapy alone after 10 weeks (20).

Individualized moderate to high intensity aerobic (70% HR) and strength training (17–20 RPE) exercise sessions for 2 times per week in 50 minute sessions including endurance and strength training were conducted in 54 high-grade glioma patients for 16 weeks in a single-arm clinical trial, the results showed improved cardiorespiratory fitness including physical work capacity, peak oxygen uptake, power output and improved QoL with larger gain in first 8 weeks (45).

In an exploratory intervention study, three groups including patients with post-aneurysmal subarachnoid hemorrhage, meningioma and healthy patients were studied, an individualized exercise program with standard energy expenditure of 17.5 kcal/kg per week was implemented for 12 weeks which showed decreased depression, insomnia and perceived stress in meningioma patients (46).

*Grabenbauer* et al. (2016) in their controlled prospective feasibility trial, studied individually tailored exercise performed for 3 times per week in 30-60-minute sessions with increased intensity in adult patients with cancer including three brain tumor patients. This study was conducted for 12 months with an adherence of 82% (47). The results showed a decrease in median Body Mass Index (BMI) and total fat mass with a significant improvement in QoL and median peak O_2_ consumption (47).

In a cross-sectional observational study including patients with high-grade recurrent glioma, patients self-reported their exercise behavior which were then correlated with functional capacity assessed by the 6-minute walk test (6MWT), QoL and performance status in the form of Karnofsky performance score (KPS). The results showed a significantly positive correlation between 6MWT and KPS as well as 6MWT and QoL, with overall improvement in performance score and QoL (48).

Overall, Individualized or self-reported exercise interventions showed improvements in physical fitness and QoL in most studies, while cognitive performance was rarely assessed. Fatigue outcomes were mixed, with mental fatigue decreasing in some studies but general fatigue showing minimal change. Feasibility and adherence were generally high. No significant safety concerns were reported, though small numbers of brain tumor patients limit generalizability of these findings.

## 4 Discussion

**Figure 4:**
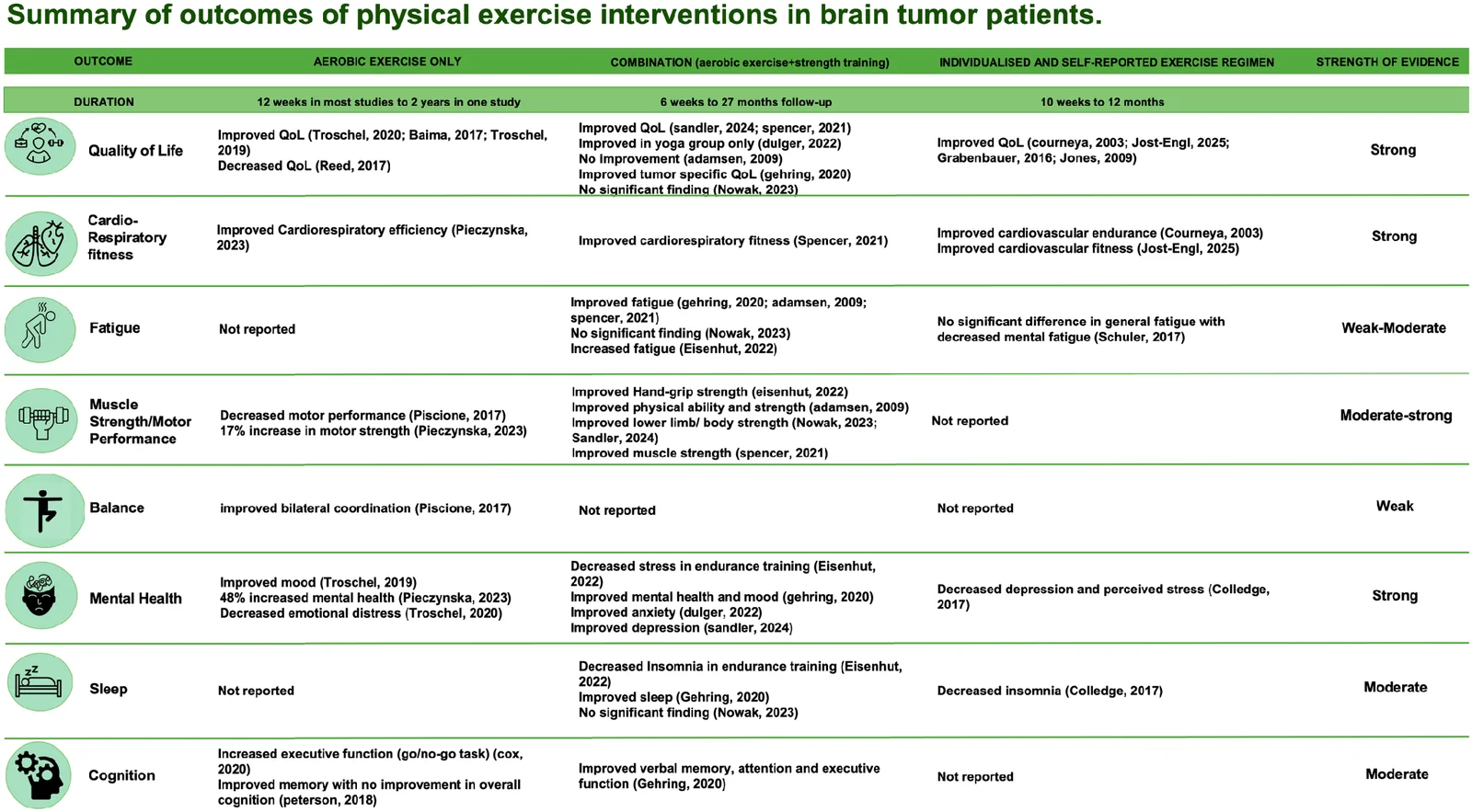
Summary of outcomes of physical exercise interventions in brain tumor patients.

Although physical exercise has been examined in patients with various types of brain tumors, the interventions, durations, and outcomes assessed have been highly heterogeneous. This systematic review synthesizes the available evidence while underscoring the ongoing challenges in translating these findings into standardized guidelines for integration into the management of patients with brain tumors. The included studies comprised both interventional studies evaluating structured exercise programs and observational studies assessing general physical activity behavior, including self-reported measures, which are more susceptible to confounding. Accordingly, these findings were interpreted with consideration of their methodological differences. Overall, the studies reviewed provide evidence that physical exercise is feasible and overall safe, with potential benefit for a number of clinical outcomes (**Figure 4**). Aerobic exercise interventions - in the form of running, cycling, walking, skiing, and soccer - were the most extensively studied interventions in group settings, at-home or supervised. Aerobic exercise showed promising results both individually and collectively with resistance or strength training. Overall, the QoL outcome showed the most disparity, with improvement after intervention in most of the studies except the observational feasibility study by *Reed* et al. (2017) that showed significant decrease in QoL after cycling exercise while no significant effect reported in others (29, 33, 39). Cardiopulmonary or aerobic fitness improved after implementing aerobic exercise alone as well as with other interventions. Physical functioning in the form of upper and lower extremity strength, flexibility and co-ordination also showed positive results in the majority of the studies with below average motor performance in a controlled cross-over clinical trial (26). *Peterson* et al. (2018) reported comparatively improved memory and verbal fluidity in the control group while *Dulger* et al. (2022) reported significantly improved cognition in both aerobic plus strength training and yoga group (22, 36). Other outcomes including fatigue, sleep quality and mental health such as mood, anxiety and depression also improved with the exception of no significant improvement reported in one pilot study (39).

In the studies that reported adherence (7 studies – 6 with mixed interventions and 1 with aerobic exercise), adherence to the prescribed exercise intervention was high, ranging from 80% to 100%. In the studies reviewed, aerobic exercise, including high-intensity exercise, was found to be feasible with reported adverse events (dizziness, back pain, leg pain, headache, joint pain, fatigue, falls and palpitations) generally matching those reported by healthy individuals participating in aerobic exercise (49). Major events reported included one episode of seizure after high intensity cardiovascular and resistance training (34). Although the data on exercise interventions for brain tumor patients are still limited, these findings suggest that the potential improvement in physical function and QoL outcomes may outweigh the rare adverse events reported. Nevertheless, the available evidence remains limited, and the apparent benefits observed across studies may partly reflect differences in baseline patient characteristics rather than the effects of exercise alone. Larger randomized controlled trials are needed to better define the balance between benefits and risks.

Although direct comparison and concise results from all the studies cannot be compiled due to different types of aerobic exercise for a varying duration, taken together, these findings suggest that aerobic exercise interventions may provide clinically meaningful benefits for selected brain tumor patients. However, given the methodological limitations, small sample sizes, and heterogeneity of the included studies, these findings should be interpreted cautiously. Therefore, current evidence does not establish that aerobic exercise independently improves clinical outcomes in brain tumor patients.

A major limitation in providing conclusive evidence of the outcomes is that in a substantial number of studies there are no specific interventions selected; instead patients were provided with individualized exercise programs in different set-ups from home-based exercise recommendations to supervised in-person interventions. Additionally, some studies evaluated self-reported exercise habits and correlated with the desired outcomes. Such self-reported measures may be subject to recall and reporting bias, which can affect the accuracy of the observed associations. However, two studies quantified exercise by average MET hours per week and a standardized energy expenditure of 17.5 kcal/kg/week, and correlated outcomes with these measures to provide a more balanced and comparable spectrum of results (37, 46).

Overall, studies with personalized exercise programs reported an improvement in QoL and fatigue with no significant difference in tumor specific QoL compared to control group with active lifestyle as reported by Gehring et al. (2020) and no significant difference in general fatigue compared to control with no physical exercise as reported by *Schuler* et al. (2017) (21, 35). Mental health components including mood, anxiety, depression and mental fatigue along with cognitive elements such as attention, verbal memory and executive function also showed improved results. *Gehring* et al. (2020) however, reported improved sustained attention in the control group (35).

Median survival also improved significantly when correlated with increased MET hours/day exercise (37). However, these findings are based on observational associations and may be subject to confounding. Furthermore, reverse causality cannot be excluded. Patients with better performance status, lower disease burden, fewer neurological deficits, or a more favorable prognosis are inherently more likely to participate in and sustain higher levels of physical activity. Consequently, it remains uncertain whether increased physical activity contributes to improved survival or whether patients with inherently better prognostic characteristics are simply more able to exercise. Therefore, these results should be interpreted with caution and do not establish a causal relationship between exercise and survival.

Adherence was reported in one study to be 82% at 12 months of intervention with reported minor adverse events related to intervention except anal discomfort reported in a patient with a history of anal cancer and progression of lymphoma in one patient (47). Another limitation, and an area for future research, is that many studies did not include a control group of patients who either did not exercise or maintained normal activity levels, which limits the ability to attribute outcomes directly to the intervention.

Yoga for 12 weeks showed overall improvement in QoL with decreased cancer-specific symptoms and sleep disturbances. In a study comparing Yoga with aerobic plus strength training, the yoga group showed improved QoL, a positive outcome which was not noted in the other intervention group (36, 44). Symptom specific Interventions were also studied. Significant improvement in balance was reported with balance and co-ordination exercises in pediatric patients with deficits secondary to brain tumor or resection. However, no significant difference in functional independent scales was reported for these children between intervention and control groups (19, 23).

Despite the differences in type and approach of intervention, all the studies had similar limitations and features that make the results less applicable in real practice and highlight the need for large scale clinical trials. Most of the studies had a small sample size which limits the generalizability and statistical power of their findings. In some studies, the author attributed that some outcomes such as intervention-related fatigue cannot be eliminated due to shorter duration of intervention and no long-term follow-up (22, 23, 41). Another disparity among the studies was the setting in which the intervention was performed. In some studies, patients were provided with education and carried out exercises at home, while in others, patients attended in-person sessions and performed supervised exercises. In certain trials, participants exercised individually, whereas in others they worked in groups, which may have enhanced consistency and adherence. Importantly, several studies implemented highly supervised or structured exercise programs within controlled research settings. While these interventions demonstrated good feasibility and adherence, their translation into routine clinical practice may be challenging, particularly in settings with limited access to specialized supervision, resources, or multidisciplinary support. Some interventions were accompanied by detailed instructions delivered through videos or live demonstrations, while others provided only general recommendations, leading to variability in how the exercise programs were implemented and followed. Overall, none of the studies that evaluated feasibility reported lack of feasibility. However, many participants approached in the initial recruitment phases of several studies declined to participate, providing evidence that there is reluctance to participate in exercise among the brain tumor patient population (28, 35, 36, 48).

In summary, the studies reviewed provide evidence that physical exercise is feasible and safe with preliminary evidence for potential clinical benefit, however, implementing exercise interventions in the clinical care of brain tumor patients remains challenging. A qualitative study evaluated the perception of caregivers, glioblastoma patients and oncology care providers on their conversations about physical exercise and reported barriers such as healthcare providers not feeling adequately trained and having limited time during visits, some clinicians considering this practice outside their scope and concern about patient safety (50). However, this study also reported that the patients were open and receptive, trusted their providers and shared positive experiences. Additionally, availability of structured programs, research studies and social groups encouraged the patients’ involvement thus, highlighting the crucial need of designing exercise interventions for brain tumor patients (50).

In addition to these implementation challenges, inter-individual variability in response to physical activity is heavily influenced by an individual’s unique genetic makeup, a concept often described in literature as “trainability.” Even when patients adhere to identical exercise prescriptions with the same FITT (Frequency, Intensity, Time, and Type) parameters, their physiological outcomes—such as gains in maximal oxygen uptake (VO_2_max), muscle hypertrophy, or metabolic health—can differ vastly (51, 52). This discrepancy is driven by specific genetic polymorphisms and mitochondrial DNA variants that dictate how the body repairs tissue and adapts to stress. For instance, the VEGFA rs2010963 polymorphism has been linked to superior adaptations in resistance training over endurance training (53), while certain mitochondrial DNA haplogroups are associated with a “low-responder” status in endurance protocols (54). Consequently, while one patient may see rapid cardiovascular improvements, another with a different genetic profile might experience minimal change from the same workload (55).

The beneficial effects of physical exercise observed across the included studies may be mediated by several emerging biological mechanisms. Exercise promotes neuroplasticity by increasing the expression of neurotrophic factors, particularly brain-derived neurotrophic factor (BDNF), as well as insulin-like growth factor-1 (IGF-1) and vascular endothelial growth factor (VEGF), which support neuronal survival, synaptic plasticity, neurogenesis, and cognitive function. In addition, regular physical activity attenuates chronic neuroinflammation through modulation of immune signaling and reduction of pro-inflammatory cytokines while stimulating the release of exercise-induced myokines and exerkines, including irisin and interleukin-6 (IL-6), which facilitate muscle-brain communication and may contribute to neuroprotection. Although these mechanisms have been demonstrated primarily in preclinical and non-neuro-oncology populations, they provide a biologically plausible explanation for the improvements in cognition, fatigue, mood, and quality of life observed across several studies included in this review. Future mechanistic studies in patients with brain tumors are warranted to determine how these pathways influence treatment response and functional recovery (56–58).

This review is strengthened by its comprehensive search strategy, adherence to PRISMA guidelines, and the inclusion of diverse study designs, offering a broad perspective on the current evidence regarding physical exercise in brain tumor patients. While most studies demonstrated positive outcomes and high adherence, the small sample sizes, heterogeneous interventions, and lack of standardized control groups limit the strength of the evidence. Feasibility outcomes were variably reported across studies, and only a subset used structured metrics. This heterogeneity limited the ability to perform a fully standardized synthesis of feasibility data. Another limitation of this review is the inclusion of heterogeneous study designs, ranging from clinical trials to case reports. While this approach was necessary due to the limited available evidence, it may reduce the overall strength and generalizability of the conclusions. Some included studies enrolled mixed cancer populations and did not report separate outcomes for brain tumor patients. Therefore, overall study results were included, and these findings should be interpreted with caution when applied specifically to the brain tumor population.

The findings should be interpreted with caution due to heterogeneity across included studies, including variation in patient populations (e.g., adult and pediatric patients, different tumor subtypes such as primary brain tumors and metastases) which may limit comparability and generalizability, as well as the high risk of bias in the studies assessed. Readers should be aware that, for example, benefits observed in pediatric patients might not directly translate to adult patients. In addition, statistically significant improvements in the outcome measures evaluated in specific studies might not readily translate into a clinical benefit for patients. Future adequately powered randomized controlled trials with adjustment for important prognostic variables, including baseline functional status and disease severity, are required to determine whether exercise independently improves survival and other clinical outcomes in this patient population. Given these uncertainties, at this time physical exercise can be safely encouraged as a supportive measure (59), but the development of structured, evidence-based guidelines for brain tumor patients will require larger, population-specific clinical trials to enable optimal integration of exercise interventions into standard neuro-oncology care.

## Data Availability

The raw data supporting the conclusions of this article will be made available by the authors, without undue reservation.
