# Supplementary material for: Physical exercise in brain tumor patients: a systematic review
Source: Front Oncol. 2026 Sep 7;16:1810188. doi: 10.3389/fonc.2026.1810188 (PMC13590378; doi:10.3389/fonc.2026.1810188)
Supplement: Supplementary file 1 [file DataSheet1.docx]

### **Supplementary Information – Search Strategy**

### **Database: PubMed**

**Date of search:** December 9, 2025

**Search strategy:**

(brain neoplasms OR brain tumor OR CNS tumor OR glioma OR high-grade glioma OR glioblastoma OR IDH-mutant glioma OR metastases OR meningioma OR primary CNS lymphoma OR PCNSL) AND (exercise OR physical exercise OR yoga OR physical activity OR ski-based exercise)

**Notes:**

- The search was conducted using free-text keywords and Boolean operators (AND, OR).
- Controlled vocabulary (MeSH terms) was not used.
- No filters were applied.

### **Database: Cochrane Library**

**Date of search:** December 9, 2025

**Search strategy:**

(brain neoplasms OR brain tumor OR CNS tumor OR glioma OR high-grade glioma OR glioblastoma OR IDH-mutant glioma OR metastases OR meningioma OR primary CNS lymphoma OR PCNSL) AND (exercise OR physical exercise OR yoga OR physical activity OR ski-based exercise)

**Notes:**

- The same core search terms were used as in PubMed, adapted for the Cochrane Library interface.
- Controlled vocabulary was not used.
- No additional filters were applied.
